# The Entomopathogenic Fungi *Metarhizium anisopliae* and *Beauveria bassiana* for Management of the Melon Fly *Zeugodacus cucurbitae*: Pathogenicity, Horizontal Transmission, and Compatability with Cuelure

**DOI:** 10.3390/insects13100859

**Published:** 2022-09-21

**Authors:** Susan K. Onsongo, Samira A. Mohamed, Komivi S. Akutse, Bernard M. Gichimu, Thomas Dubois

**Affiliations:** 1 International Centre of Insect Physiology and Ecology (*icipe*), Nairobi 00100, Kenya; 2 Department of Agricultural Resource Management, University of Embu, Embu 60100, Kenya

**Keywords:** *Beauveria bassiana*, compatibility, horizontal transmission, *Metarhizium anisopliae*, mortality, *Zeugodacus cucurbitae*

## Abstract

**Simple Summary:**

The melon fly *Zeugodacus cucurbitae* is an invasive fruit fly that causes extensive damage to many fruit crops. Sustainable management of this pest can be achieved through environmentally friendly and safely integrated pest management (IPM), in which the use of biopesticides is a key component. In this study, various isolates of entomopathogenic fungi were evaluated on two developmental stages of this pest. The fungal isolate ICIPE 69 has the greatest potential. The study also evaluated whether ICIPE 69 could be used together with the male pheromone of the melon fruit fly, as well as whether infected flies could contaminate clean flies. Our study indicates that ICIPE 69 can be included in IPM packages to reduce yield losses.

**Abstract:**

In the laboratory, the pathogenicity of thirteen isolates of *Metarhizium* *anisopliae* (Metschnikoff) Sorokin and two isolates of *Beauveria bassiana* (Balsamo) Vuillemin against the melon fly *Zeugodacus cucurbitae* (Coquillett) were assessed by exposing adults to 0.3 g of dry conidia (~3 × 10^9^ conidia) of each isolate for 5 min and monitoring mortality for up to 5 days. Compatibility with a male pheromone, cuelure, (4-(p-acetoxyphenyl)-2-butanone), was determined by testing conidial germination and germ tube growth of the most promising isolate, *M. anisopliae* ICIPE 69, in the presence of cuelure at different temperatures. For horizontal transmission, the flies were separated by sex, separately exposed to *M. anisopliae* ICIPE 69, and subsequently mixed with non-exposed flies from the other sex. The most pathogenic isolates were *M. anisopliae* ICIPE 69, 18, and 30, causing mortalities of 94, 87, and 81%, with 5 days post-exposure, respectively. *Metarhizium anisopliae* ICIPE 69 caused the highest pupal mortality of 74%, with 15 days post-exposure. Horizontal transmission of *M. anisopliae* ICIPE 69 among male and female *Z. cucurbitae* was confirmed by 59 and 67% mortality after exposure to infected donor males and females, respectively. *Metarhizium anisopliae* ICIPE 69 affected the oviposition, but not hatchability, of infected *Z. cucurbitae* females. *Metarhizium anisopliae* ICIPE 69 is, therefore, a potential isolate for biopesticide development for *Z. cucurbitae* management in cucurbit production systems.

## 1. Introduction

Cucurbits are widely cultivated around the world, mainly in the tropics and subtropics [1]. China is the largest producer in the world, with an annual production at >139 million tonnes, representing 79.7% of the world production [2]. Cucurbits are both a source of income, as well as a nutritious food [3], with the fruits, leaves, and seeds consumed as good sources of vitamins and minerals [4,5]. Cucurbits are ranked among the major vegetables grown in Kenya for local consumption and export [6]. Their production is, however, constrained by various pests, especially Tephritid fruit flies [7,8]. Among these, the melon fly *Zeugodacus cucurbitae* (Coquillett) (Diptera: Tephritidae) is the most destructive and widespread species that affects cucurbit production [9]. *Zeugodacus cucurbitae* is a highly polyphagous pest that attacks a wide range of plants, with a preference for Cucurbitaceae [10,11,12]. The pest also has high dispersive potential and reproductive rates [12,13,14]. Sexually mature females cause direct losses by ovipositing eggs under the skin of the fruits with hatched larvae feeding inside the fruit, leading to rotting [15]. The pest causes yield losses of 30–100%, depending on environmental factors and cucurbit species, leading to loss of food, income, and employment for many households in sub-Saharan Africa [13].

Over the years, cucurbits production in Kenya has been facing challenges [16], and this has been partly attributed to infestation by *Z. cucurbitae* and other pests [17]. Farmers mostly use harmful chemical insecticides (primarily profenofos, cypermethrin, imidacloprid, and cyflutrin) to control *Z. cucurbitae*, but their intensive use has negative effects on the environment and may result in the development of pesticide resistance [18,19,20]. Integrated pest management (IPM) approaches, such as the use of protein baits, sterile insect technique, parasitoids, and entomopathogenic fungi, are being developed and commercialized as alternatives [21,22,23,24,25,26].

Due to their high mobility, the management of the adult stage of *Z. cucurbitae* can be optimized by using auto-dissemination devices for an ‘attract-and-infect’ approach. The approach works by attracting adult flies to a device that is baited with entomopathogenic fungi. The flies then come in contact with the pathogen and, upon return to the environment, disseminate the pathogen among the population through physical contact [27]. The approach has been effective for other fruit fly species, such as the mango fruit fly *Ceratitis cosyra* (Walker) (Diptera: Tephritidae), oriental fruit fly *Bactrocera dorsalis* (Hendel) (Diptera: Tephritidae) [26], and Mediterranean fruit fly *Ceratitis capitata* (Wiedemann) (Diptera: Tephritidae) [28]. In a recent study by Faye et al. [29] on *Bactrocera dorsalis*, this approach reduced the population of the pest by up to 90%. The pheromone cuelure (4-(p-acetoxyphenyl)-2-butanone) attracts male adults of *Z. cucurbitae* and can, thus, be used together with entomopathogenic fungi in an attract-and-infect approach. The success of this approach is, however, dependent on factors such as the virulence and persistence of the entomopathogenic fungi, ability of the auto-dissemination device to attract the target insect and transmit inoculum, and, most importantly, compatibility of the pheromone with entomopathogenic fungi [28,30].

Most studies conducted in Kenya on Tephritid fruit flies have used *Metarhizium anisopliae* (Metschnikoff) Sorokin (Hypocreales: Clavicipitaceae) and *Beauveria bassiana* (Balsamo) Vuillemin (Hypocreales: Cordycipitaceae), and the results have shown *M. anisopliae* to be the most effective on different fruit fly species, in terms of the percentage of mortality and lethal time (LT) values [31,32]. However, the pathogenicity of locally available entomopathogenic fungi to *Z. cucurbitae* has not yet been studied. Therefore, the main purpose of this study was to evaluate the virulence of 13 isolates of *M. anisopliae* and two isolates of *B. bassiana* against puparia and adults of *Z. cucurbitae*. Subsequently, the compatibility of the most effective isolate, with a commercially available lure, based on cuelure, was assessed. In addition, the study analyzed the effectiveness of horizontal transmission of the most virulent fungal isolate and its effects on reproduction of infected female *Z. cucurbitae*.

## 2. Materials and Methods

### 2.1. Insect Source and Rearing Conditions

Adult *Z. cucurbitae* were obtained from incubated cucurbit fruits collected from Nguruman, Kenya. A mass-reared colony was maintained on winter squash *Cucurbita moschata* Duch. (Violales: Cucurbitales) at the International Centre for Insect Physiology and Ecology (*icipe*), Nairobi, Kenya, and the 3rd generation was used in our experiments. To boost the colony and obtain young flies of the same age, they were exposed to *C. moschata* for 24–48 h for oviposition. A plastic container (35 × 20 × 12 cm), containing sterilized sand up to a depth of 5 cm and a wire mesh placed at 15 cm above, was used to hold the infested *C. moschata*. After 10 days of incubation, 3rd instars emerged from infested *C. moschata* and fell onto the sand to pupate. The larvae were collected for the pupation experiment, and the rest were left to pupate. The puparia were collected in 90 mm diameter plastic Petri dishes and placed in Perspex cages (15 × 15 × 15 cm) for adults to emerge. The emerged adult flies were maintained on a sugar and yeast hydrolysate-based artificial diet with 10 mL water in falcon tube lids filled with pumice granules [33] at a relative humidity (RH) of 45%, temperature of 27 ± 2 °C and photoperiod of L12/D12 h.

### 2.2. Fungal Sources and Maintenance of Fungal Cultures

Thirteen isolates of *M. anisopliae* and two isolates of *B. bassiana* were obtained from *icipe*’s germplasm repository and had been preserved at −80 °C prior to use (Table 1). The isolates were selected based on their potency towards various pests, including Tephritid fruit flies. The *M. anisopliae* and *B. bassiana* isolates were revived by culturing them on Sabouraud dextrose agar (SDA) (40 g dextrose, 10 g peptone, 15 g agar, and 1 L water) (Oxoid, Basingstoke, UK) and potato dextrose agar (PDA) (200 g potato infusion, 20 g dextrose, 20 g agar, and 1 L water) (Oxoid), respectively, at 26 ± 2 °C, in complete darkness, for 21 days. Fungal isolates harvested from the 21-day-old fungal cultures from different Petri dishes were uniformly mixed. Conidial viability was tested by taking a sample from the fungal cultures and suspending the inoculum in 10 mL of sterile 0.01% Triton in a 30 mL universal bottle containing glass beads measuring 3 mm in diameter. The conidial suspension was vortexed for 3 min at 700 rpm to attain homogeneity, from which a final concentration of 3 × 10^6^ conidia/mL was prepared using an improved Neubauer hemocytometer under a light microscope (LEICA DM 2000, Leica Microsystems, Morrisville, NC, USA) at 40× magnification [34]. A volume of 0.1 mL of conidial suspension was then spread onto sterilized SDA or PDA in Petri dishes. Each isolate was cultured in three Petri dishes to act as replication in a completely randomized design (CRD) and tested for viability. The plates were incubated at 26 °C for 16–18 h, followed by fixing with lacto-phenol cotton blue (Millipore Corporation, Billerica, MA, USA) to terminate fungal growth. Sterile slide coverslips (2 × 2 cm) were placed on the top of each Petri dish and viability was recorded from each Petri dish using a compound microscope (LEICA DM 500). Viability was determined by counting a total number of 100 conidia for both germinated and non-germinated conidia per coverslip [35]. Conidia were deemed to have germinated when the length of the germ tube was at least twice the diameter of the conidium. Percentage germination per coverslip was equal to the number of germinated conidia [36].

### 2.3. Virulence of Entomopathogenic Fungal Isolates to Zeugodacus cucurbitae Adults

The bioassay was set in the laboratory following a CRD with five replicates per isolate. A mass of 0.3 g dry conidia spores of each isolate was harvested, as described in Section 2.2, weighed using a weighing balance (Wincom Company, Changsha, Hunan, China), and evenly spread onto a sterile contaminating device using a spatula. The contaminating device was a cylindrical plastic vial measuring 9.5 × 4.8 cm, with the inside lined with a velvet fabric and white netting at the bottom. Twenty young (aged 5–7 days) flies were randomly picked from the colony and allowed to walk on the velvet fabric of the contaminating device for 5 min using a procedure described by Qazzaz et al. [37], while uninoculated insects acted as a control and were exposed to contamination devices that had not been inoculated. The inoculated and control flies were then transferred into 15 × 15 × 15 cm clean Perspex cages supplied with ~10 mL water in falcon tube lids filled with pumice granules and artificial adult food (dry mixture of 3:1 sugar:enzymatic yeast hydrolyzate) in a Petri dish and maintained at the conditions mentioned in Section 2.1. Insect mortality was recorded daily for 5 days. Dead insects from each treatment were surface sterilized in 70% EtOH and 2.5% NaOH for 2–3 min, rinsed thrice in sterile distilled water, and transferred into Petri dishes lined with damp sterilized Whatman filter paper to allow for mycosis. Mycosis was recorded from incubated cadavers after 2–5 days by observing any fungal outgrowth using a microscope. Mortality due to fungus was confirmed through the presence of green and white-colored mycelium for *M. anisopliae* and *B. bassiana*, respectively, on the surface of the cadavers, and identification of entomopathogenic fungi, which was established by comparing with mother cultures. If in doubt, slides were prepared from mycelial outgrowth and conidia to confirm fungal identity.

### 2.4. Effect of Selected Metarhizium anisopliae Isolates on Zeugodacus cucurbitae Puparia Eclosion

The experiment was set up in the laboratory following a completely randomized design (CRD), and each treatment was replicated four times. *Zeugodacus cucurbitae* larvae were collected from laboratory-infested *C. moschata* fruits, as described in Section 2.1, and placed into sterile 90 mm diameter Petri dishes prior to the experiment. Fungal suspensions of 1 × 10^6^, 1 × 10^7^, and 1 × 10^8^ conidia/mL were made in 0.1% Tween 100 from the fungal isolates (*M. anisopliae* ICIPE 69, ICIPE 18 and ICIPE 30) that were most virulent based on the adult pathogenicity experiments. For control treatments, 0.1% Tween 100 solution was used without any fungal conidia. A volume of 20 mL suspension was then sprayed evenly using a 500 mL hand sprayer on 100 g of sterile (autoclaved at 121 °C for 1 h) clay loamy soil placed in 15 × 15 × 15 cm Perspex cages. Fifty 3rd instars, which is the last instar before pupation, were introduced into the sterile soil of each cage by allowing them to pop from the holding Petri dish. The experiment was monitored daily for 15 days to assess the pupation of the introduced larvae. Formed puparia and emerged adults were counted, immediately moved into new 15 × 15 × 15 cm clean Perspex cages, and provided with diet and water, as described in Section 2.1. Mortality of the emerged flies was recorded daily for 5 days, and the cadavers were removed from the Perspex cages, surface-sterilized, and placed on moist Whatman filter paper in sterile 90 mm diameter plastic Petri dishes that were sealed with parafilm. The Petri dishes were kept at 20–26 °C, 40–70% RH, and L12/D12 h and monitored daily for 4 days for mycosis, according to Section 2.3.

### 2.5. Compatibility of Metarhizium anisopliae ICIPE 69 with Cuelure

Percentage conidial germination and germ tube length were used to establish the compatibility of *M. anisopliae* ICIPE 69, which was identified as the most promising isolate for management of *Z. cucurbitae*, with cuelure (Cue-lure Plug, Farmtrack Consulting, Nairobi, Kenya). A conidial suspension of 1 × 10^7^ conidia/mL was prepared from the stock solution [34], after which 10 mL of the suspension was poured through a filter holder unit under aspirator vacuum, and the spores were retained on a 47 mm diameter and 0.45 µm pore size nitrocellulose filter membrane (Sigma Chemicals, Balcatta, Australia). The nitrocellulose filter membranes containing conidia were placed under a laminar flow cabinet for 30 min to dry and then transferred to a single-glass desiccator (2.5 L) for exposure to cuelure, following the protocol used by [38,39]. Fungus-treated nitrocellulose membranes were exposed to cuelure and sampled for viability at day 1, 2, 3, 6, and 8 post-exposure. One fungus-treated nitrocellulose filter membrane containing conidia was removed from the desiccator and transferred into sterile-titrated water (0.05% Triton X-100) for viability assessment.

The treatments were set at different temperatures of 18 °C, 25 °C, and 30 °C to test if temperature can affect the emission and diffusion of cuelure. A control without cuelure was also included, according to [39,40]. To determine percentage conidial germination and germ tube length, nitrocellulose membranes were individually removed from the desiccators, transferred into 10 mL sterile water containing 0.05% Triton X-100 in a 30 mL universal bottle, and vortexed for 3 min to dislodge conidia. A volume of 0.1 mL of 3 × 10^6^ conidia/mL suspension was prepared and spread evenly on SDA in three Petri dishes. The Petri dishes were incubated at the conditions mentioned in Section 2.5, examined after 16–18 h, and percentage conidial germination was determined as described in Section 2.2. The same procedure, but without cuelure, was applied for the control treatment. Germ tube length was measured using a Leica Application Suite LAS EZ V1.5.0 at 200× magnification.

### 2.6. Horizontal Transmission of Metarhizium anisopliae ICIPE 69 among Zeugodacus cucurbitae Adults

Adult flies were obtained as described in Section 2.1 and separated by sex, based on the presence of an ovipositor. The flies were inoculated as described in Section 2.3. In the first set of the experiment, five groups of 20 female adults were exposed to dry fungal spores by allowing them to walk on fungal contaminated velvet material for 5 min and each set of 20 flies was subsequently transferred to a Perspex cage. Five flies from each cage were randomly aspirated and spore retention assessed at 0, 2, 4, 6, 8, and 24 h post-exposure. The number of conidia acquired by each fly was quantified by transferring them individually into 15 mL plastic vials containing 1 mL of sterile water with 0.05% Triton X-100. Vials were vortexed for 3 min to dislodge conidia. The number of conidia obtained at the different times were estimated using an improved Neubauer hemocytometer under a light microscope at 40× magnification, according to [39,41].

To evaluate conidial transmission between flies, four groups of 20 10-day-old males were inoculated (donors), according to the procedure described in Section 2.3. After 24 h post-inoculation, the infected male flies were introduced to 20 fungus-free, 7-day-old females (recipients) and held together in Perspex cages to allow for contact. A similar set of experiments were conducted with female flies as the donors and male flies as the recipients. Another group of 20 fungus-free males and females were also held together under similar conditions and used as controls. Each experiment was replicated four times. After 24 h of contact, the flies were separated by sex and held in separate perplex cages for 5 days at 20–26 °C, 40–70% RH, and L12/D12 h, where their mortality was recorded daily. Mycosis was assessed on the cadavers, as described in Section 2.3.

To measure reproduction potential, 20 fungus-infected 7-day-old female flies were put in Perspex cages together with 20 fungus-free 7-day-old male flies and held together for 5 days, while provided with artificial diet and water, as described in Section 2.1. *Cucurbita moschata* epicarp (cut in spheres), with a diameter of 3 cm, was used as substrate for oviposition. The experiment was set in a CRD and replicated four times. Oviposited eggs were counted daily for 5 days under a dissecting microscope (Leica EZ4HD). Twenty eggs in each treatment were randomly picked daily and transferred into Petri dishes lined with a damp black cloth. Petri dishes were incubated and the number of eggs that hatched were recorded daily for 5 days. Eggs that did not hatch after 5 days were considered non-viable and dead.

### 2.7. Data Analysis

All data sets were checked for normality before analysis. Adult fly mortality was adjusted for natural mortality using Abbott’s formula [42]. Data on percentage conidial germination, puparia eclosion, spore retention, and percentage fly mortality were subjected to a generalized linear model (GLM), assuming a binomial distribution and logit link. Data for estimation of lethal time (LT_50_) were analyzed using a generalized linear model, assuming a binomial distribution and probit link (probit model). LT_50_ was estimated using the ‘dose.p’ function from the MASS package of the R statistical software package (R Development Core Team [43]). Three-way analysis of variance (ANOVA) was used to analyze the effect of temperature, cuelure and time on percentage conidial germination, and germ tube length. Egg oviposition data was log-transformed and analyzed using ANOVA. When statistically significant, means were compared using the Student–Newman–Keuls (SNK) tests. All data analyses were performed using R version 4.1.0.

## 3. Results

### 3.1. Pathogenicity and Virulence of Entomopathogenic Fungal Isolates against Zeugodacus cucurbitae Adults

The percentages of conidial germination varied significantly among the fungal isolates (χ^2^ = 70.89 df = 44; *p* < 0.0001) and ranged from 87.25 to 97.82% (Table 2). The effect of the fungal isolates on adult fly mortality was highly significant (χ^2^ = 2884.60; df = 14; *p* < 0.0001), ranging from 13.50 to 91.42%. The two *B. bassiana* isolates (ICIPE 270 and ICIPE 603), which recorded the lowest mortality rates, did not differ significantly from each other in both percentage conidial germination and induced fly mortality. *Metarhizium anisopliae* ICIPE 69 caused the highest mortality rate at 91.42%. The LT_50_ values varied from 3.84 to 4.83 days. *Metarhizium anisopliae* ICIPE 69 and ICIPE 18 took the shortest time (3.84 and 3.96 days, respectively) to cause 50% fruit fly mortality. Based on percentage fly mortality and LT_50_ values, *M. anisopliae* isolates ICIPE 69, ICIPE 18, and ICIPE 30 outperformed all other isolates and were selected for further evaluation to assess their effectiveness in reducing *Z. cucurbitae* puparia eclosion.

### 3.2. Effect of Selected Metarhizium anisopliae Isolates on Zeugodacus cucurbitae Puparia Eclosion

There was a significant difference of eclosion between puparia in the control treatment and those maintained on inoculated soil (χ^2^ = 708.67; df = 1; *p* < 0.0001) (Table 3). Within soils treated with entomopathogenic fungi, conidial concentration (χ^2^ = 299.20; df = 2; *p* < 0.0001), and isolate (χ^2^ = 98.84; df = 2; *p* < 0.0001) significantly affected eclosion. The concentration 1 × 10^8^ caused the lowest eclosion across isolates, while *M. anisopliae* ICIPE 69 caused the lowest eclosion across concentrations. There was also an interaction between conidial concentration and isolate (χ^2^ = 60.20; df = 4; *p* < 0.0001) on puparia eclosion, with *M. anisopliae* ICIPE 69 inhibiting puparia eclosion across lower concentrations, unlike *M. anisopliae* ICIPE 18 and ICIPE 30.

### 3.3. Mortality of Zeugodacus cucurbitae Adults Eclosed from Inoculated Soil

Mortality was significantly higher (χ^2^ = 202.79; df = 1; *p* < 0.0001) from puparia maintained on inoculated soil, compared to those in control treatments (Table 4). Conidial concentration (χ^2^ = 275.07; df = 2; *p* < 0.0001) and isolate (χ^2^ = 39.62; df = 2; *p* < 0.0001) significantly affected the percentage mortality of emerged adults. There was also an interaction between the fungal isolates and conidial concentration (χ^2^ = 156.06; df = 4; *p* < 0.0001). The three tested isolates caused fly mortality ranging from 20.23 to 74.29%, depending on conidial concentration. The highest conidial concentration of 1 × 10^8^ conidia/mL induced the highest mortality for *M. anisopliae* ICIPE 69, while, for the concentration of 1 × 10^7^ conidia/mL, the highest mortality was recorded with *M. anisopliae* ICIPE 30.

### 3.4. Compatibility of Metarhizium anisopliae ICIPE 69 with Cuelure

Percentage conidial germination of *M. anisopliae* ICIPE 69 was significantly affected by temperature (χ^2^ = 160.85; df = 2; *p* < 0.0001), presence of cuelure (χ^2^ = 121.41; df = 1; *p* < 0.0001), and time of exposure (χ^2^ = 441.05; df = 4; *p* < 0.0001) (Table 5). There was an interaction between the exposure time and cuelure (χ^2^ = 88.76; df = 4; *p* < 0.0001), as well as between the cuelure and temperature (χ^2^ = 71.83; df = 8; *p* < 0.0001), on the percentage of conidial germination. Cuelure significantly reduced percentage conidial germination at the lowest temperature (18 °C) at the start of exposure, while it significantly reduced percentage conidial germination at the higher temperatures (25 °C and 30 °C) at the end of exposure. The germ tube length of the *M anisopliae* ICIPE 69 isolate was significantly affected by temperature (F = 14.59; df = 2105; *p* < 0.0001), presence of cuelure (F = 63.92; df = 1105; *p* < 0.0001), and time of exposure (F = 41.28; df = 4; *p* < 0.0001) (Table 6). There was an interaction between cuelure and temperature (F = 3.46; df = 2; *p* < 0.0001) on the germ tube length. A significant reduction in the germ tube length was observed in all the treatments over time, but was more pronounced at 25 °C and 30 °C.

### 3.5. Horizontal Transmission of Metarhizium anisopliae Inoculum

There was a significant (F = 3.345; df = 5, 24; *p* = 0.0197) difference in spore acquisition and retention at different monitoring hours. The number of acquired spores were 14.41 × 10^5^ (±3.27), 12.45 × 10^5^ (±1.33), 12.55 × 10^5^ (±2.24), 9.45 × 10^5^ (±2.11), 6.55 × 10^5^ (±1.62), and 6.41 × 10^5^ (±0.87) at 0, 2, 4, 6, 8, and 24 h, respectively. Adult fly mortality varied between donors and recipients (χ^2^ = 640.32; df = 3; *p* < 0.0001) (Table 7). In donors, 100% mortality was observed, with lower lethal time values, compared to the recipients.

**Figure 1:**
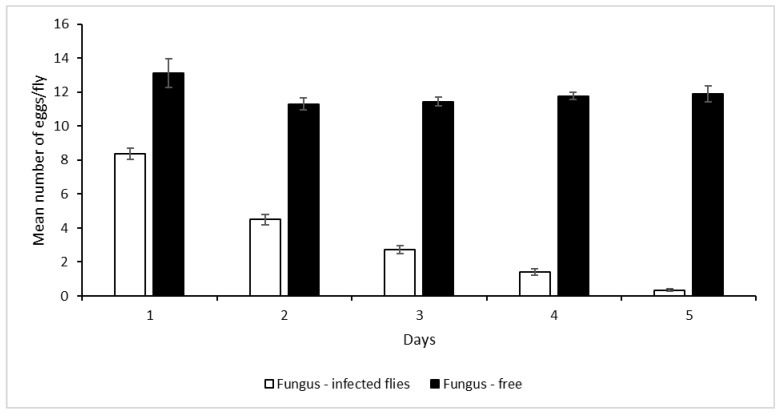
Effect of *Metarhizium anisopliae* ICIPE 69 on oviposition of *Zeugodacus cucurbitae*.

The ability of *Z. cucurbitae* females to oviposit eggs was significantly (χ^2^ = 144.86; df = 9; *p* < 0.0001) reduced by *M. anisopliae* ICIPE 69 infection (Figure 1). More eggs were laid by fungus-free female flies than by fungus-infected ones in all days. However, there was no significant (F = 0.695; df = 1, 6; *p* =0.695) difference in the hatchability of the eggs oviposited by the fungus-treated flies and untreated flies, which averaged 15.0 and 15.4 eggs for fungus-infected flies and fungus-free flies, respectively, for the five days.

## 4. Discussion

The study aimed at identifying an efficient fungal biopesticide for the management of *Z. cucurbitae*, which is a devastating pest of cucurbits. Results from the screening bioassays of *M. anisopliae* and *B. bassiana* isolates against 3rd instars and adults of *Z. cucurbitae* indicated that all the tested isolates were pathogenic to the pest, with *M anisopliae* ICIPE 18, ICIPE 30, and ICIPE 69 causing the highest mortality. Susceptibility of other Tephritid fruit flies to these entomopathogenic fungi has been reported in other studies. For example, *B. bassiana* has been found to be virulent against different life stages of *C. capitata* [36,44,45], *B. dorsalis* [46], and the peach fruit fly *Bactrocera zonata* (Saunders) (Diptera: Tephritidae) [47]. *Metarhizium anisopliae* has been reported to be effective against *B. zonata* [47].

In this study, *B. bassiana* isolates were found to be less virulent, compared to *M.* *anisopliae*. This is consistent with previous findings reported for the fruit fly species, such as for the Natal fruit fly *Ceratitis rosa* var. *fasciventris* Karsch (Diptera: Tephritidae), *C. capitata* [48], and *C. cosyra* [31,49]. The most virulent isolates of *M. anisopliae*, in descending order, were *M. anisopliae* ICIPE 69, ICIPE 18, and ICIPE 30. *Metarhizium anisopliae* ICIPE 18 has been reported to be virulent against *C. capitata*, *C. rosa* var. *fasciventris*, and *C. cosyra* [26]. From previous studies, *M. anisopliae* ICIPE 69 and ICIPE 30 were shown to be effective against three aphid species, i.e., the cabbage aphid *Brevicoryne brassicae* Linnaeus (Hemiptera: Aphididae), turnip aphid *Lipaphis pseudobrassicae* Davis (Hemiptera: Aphididae), and cotton aphid *Aphis gossypii* Gossypii L. (Homoptera; Aphididae) [50]. The fungal isolate *M. anisopliae* ICIPE 69 proved to be pathogenic against the Western flower thrips *Frankliniella occidentalis* Pergande (Thysanoptera: Thripidae) [38] and cowpea aphid *Aphis craccivora* Koch (Hemiptera: Aphididae) [51]. *Metarhizium anisopliae* ICIPE 69 has been commercialized by the Real IPM Company (Thika, Kenya) against some species of fruit flies, mealybugs, and thrips [21], and the findings of this study will possibly pave the way for label extension of this biopesticide towards *Z. cucurbitae*.

The difference in virulence among the isolates of *M. anisopliae* was confirmed by LT_50_ data. The lowest LT_50_ values in this study were observed in *M. anisopliae* ICIPE 69, ICIPE 18, and ICIPE 30. These LT_50_ values were slightly higher than LT_50_ values obtained by Dimbi et al. [31], with *M. anisopliae* ICIPE 18 and other *M. anisopliae* isolates against *C. capitata* and *C. rosa* var. *fasciventris*. However, they were in the same range as LT_50_ values reported by Mweke et al. [51], with some *M. anisopliae* isolates against *A. craccivora* at similar conidial concentrations. These differences in LT_50_ of the target insects could be attributed to differences in the virulence of the fungal isolates used in the different studies or differences in fungal virulence across fruit fly species.

*Metarhizium anisopliae* has also shown effectiveness in controlling the puparia eclosion of other insect pests. For instance, a study by Gul et al. [47] reported that *M. anisopliae* MA-02 suppressed the eclosion of *B. zonata* puparia maintained in soil that had been inoculated with the fungal isolate. This finding was confirmed in our study, as eclosion of flies in the fungus-free soil (control treatment) was higher than that in the *M. anisopliae* ICIPE 69-inoculated soil. The eclosion rates obtained in this study were within the range of those reported by Beris and Papachristis 2013 [48]. Comparable findings were also reported in other related studies [32,52,53,54]. Our study targeted the 3rd instars because earlier instars of *Z. cucurbitae* are concealed inside the host fruit, and only the 3rd instars pop out and drop into the soil to pupate [32]. Therefore, targeting the last instar of *Z. cucurbitae* is an adequate control strategy has been shown to result in high levels of pupal mortality.

The higher mortality of adult flies that eclosed from puparia reared in soil inoculated with entomopathogenic fungi, compared to those from non-inoculated soil, is in line with previous findings for related *Bactrocera* species [55]. Hypothetically, the adult flies that emerged from the inoculated soils acquired the inoculum after eclosion from the soil and succumbed to it, as confirmed by development of mycoses upon death. However, the duration from eclosion to death was relatively short, which could suggest that few infective propagules may have been acquired before pupation. Pupal mortality, due to *M. anisopliae* infection, was also reported by Ekesi and Maniania 2000 [56] in *F. occidentalis* and the bean flower thrips *Megalurothrips sjostedti* (Trybom) (Thysanoptera: Thripidae).

*Metarhizium anisopliae* ICIPE 69 was selected for compatibility studies with the pheromone cuelure because it had been found the most promising isolate against *Z. cucurbitae* adults at different temperature regimes [17] and was found to be effective against puparia in the present study. Cuelure was found to be incompatible with *M. anisopliae* ICIPE 69 at different temperature regimes. Fungal viability was significantly reduced by exposure to cuelure over time, since fungal growth deteriorated faster, compared to the control treatments. Similar observations were made with *M. anisopliae* ICIPE 69 by Mfuti et al. [34] and Nana et al. [40] for the attraction, aggregation, and attachment pheromone (AAAP), as well as seven other compounds used for thrips attraction. Possibly, cuelure may have some antagonistic effects to fungi, as previously reported [57,58,59]. To be effective, cuelure could be placed outside the auto-dissemination device.

For an entomopathogenic fungal isolate to be an effective biocontrol agent, its dispersal should be efficient, and the secondary infection should still cause mortality. Horizontal transmission can be achieved through avenues such as mating or physical contact. Fruit flies generally engage in extensive grooming after having been in contact with entomopathogenic fungi; therefore, the number of conidia retained on them after grooming is important for successful horizontal transmission. In this study, *Z. cucurbitae* treated with entomopathogenic fungi (donors) were able to transmit the fungus to non-contaminated ones (recipients) causing substantial secondary mortality, which was comparable to the primary mortality rate. Effective horizontal transmission of *M. anisopliae* inoculum was also reported by Opisa et al. [39] among *Spoladea recurvalis* Fabricius (Lepidoptera: Crambidae) adults, Akutse et al. [60] among tomato leafminer *Tuta absoluta* (Meyrick) (Lepidoptera: Gelechiidae) adults, and Mkiga et al. [61] among false codling moth *Thaumatotibia leucotreta* (Meyrick) (Lepidoptera: Tortricidae) adults. Among fruit fly species, Quesada-Moraga et al. [62] reported successful horizontal transmission among laboratory populations of *C. capitata*, and Dimbi et al. [41] reported successful fly-to-fly transmission in *C. cosyra*, *C. fasciventris*, and *C. capitata*. Effective horizontal transmission has also been reported for *B. bassiana* in the kissing bug *Triatoma infestans* Klug (Hemiptera: Reduviidae) [63] and Mexican fruit fly *Anastrepha ludens* Loew (Diptera: Tephritidae) [64].

This study further demonstrated the effectiveness of *M. anisopliae* ICIPE 69 to reduce the egg-laying capacity of infected female *Z. cucurbitae*. A study by Dimbi et al. [65] also demonstrated the effect of *M. anisopliae* on the mating behavior and egg-laying capacity of *C. capitata*, *C. cosyra*, and *C. fasciventris*.

## 5. Conclusions

From the findings of this study, it was evident that *M. anisopliae* is more virulent to *Z. cucurbitae* than *B. bassiana*. The most virulent isolates of *M. anisopliae* were identified as *M. anisopliae* ICIPE 69, ICIPE 18, and ICIPE 30, and the three are, therefore, recommended for further screenhouse and field studies towards IPM of *Z. cucurbitae*. This study also demonstrated that *M. anisopliae* ICIPE 69 can be effectively used to suppress the eclosion of *Z. cucurbitae* puparia and subsequently cause the mortality of the emerged adults. In the laboratory, *M. anisopliae* ICIPE 69 did not seem compatible with cuelure, necessitating further studies before the fungus could be synergistically used with cuelure for the effective control of *Z. cucurbitae* through an attract-and-infect approach. This study further demonstrated effective horizontal transmission of *M. anisopliae* ICIPE 69 among adult *Z. cucurbitae* populations, presumably increasing the efficacy of the fungus in *Z. cucurbitae* IPM programs. It is, however, important to carry out a study and establish the effectiveness of ICIPE 69 on *Z. cucurbitae* under field conditions, as well as the effect on non-target organisms.

## Figures and Tables

**Table 1:** Identities of fungal isolates tested against *Zeugodacus cucurbitae* adults.

Fungal Species	Fungal Isolate	Source	Place of Origin (Country)	Year of Isolation
*M. anisopliae*	ICIPE 7	*Rhipicephalus appendiculatus* Neumann (Ixodida: Ixodidae)	Rusinga Island (Kenya)	1996
	ICIPE 18	Soil	Mbita (Kenya)	1989
	ICIPE 20	Soil	Migori (Kenya)	1989
	ICIPE 30	Busseola fusca (Fuller) (Lepidoptera: Noctuidae)	Kendubay (Kenya)	1989
	ICIPE 62	Soil	Matete (DR Congo)	1990
	ICIPE 69	Soil	Matete (DR Congo)	1990
	ICIPE 78	*Tomobrachyta nigroplagiata Fairmaire* (Coleoptera: Cerambycidae)	Ungoe (Kenya)	1990
	ICIPE 81	*Kraussaria angulifera* (Krauss) (Orthoptera: Acrididae)	Kaffrine (Senegal)	2003
	ICIPE 315	*Tetranychus urticae* K. (Trombidiformes: Tetranychidae)	Kerugoya (Kenya)	2006
	ICIPE 655	Soil	Kabuti (Kenya)	2008
	ICIPE 656	Soil	Kapiti (Kenya)	2008
	ICIPE 674	Soil	Mariakani (Kenya)	2008
	ICIPE 690	Lepidoptera Larvae	Kenya	2010
*B. bassiana*	ICIPE 279	Coleopteran larvae	Kericho (Kenya)	2005
	ICIPE 603	Hymenoptera	Taita (Kenya)	2007

**Table 2:** Percentage conidial germination, mortality, and lethal time of entomopathogenic fungal isolates, and their pathogenicity to *Zeugodacus cucurbitae* adults at 5 days post-treatment.

Fungal Species	Isolates	Germination ± SE (%)	Mortality ± SE (%)	LT_50_ ^1^ (Days) (95% FL ^2^)
*Metarhizium anisopliae*	ICIPE 7	90.32 ± 3.84cd	56.58 ± 3.63f	4.83 (4.79–4.86)
	ICIPE 18	94.97 ± 0.67abcd	85.56 ± 2.57b	3.96 (3.94–3.98)
	ICIPE 20	95.28 ± 0.82abcd	75.63 ± 4.77c	4.39 (4.37–4.41)
	ICIPE 30	97.20 ± 0.45abc	75.87 ± 3.30c	4.12 (4.09–4.15)
	ICIPE 315	94.21 ± 1.75abcd	55.74 ± 4.34f	4.83 (4.80–4.87)
	ICIPE 690	94.28 ± 1.65abcd	42.53 ± 2.71g	–
	ICIPE 62	90.23 ± 0.56cd	71.24 ± 4.19cd	4.40 (4.37–4.43)
	ICIPE 655	92.17 ± 2.98abcd	56.66 ± 3.94f	4.87 (4.83–4.91)
	ICIPE 656	90.46 ± 1.33abcd	62.74 ± 5.98ef	4.73 (4.70–4.77)
	ICIPE 674	95.56 ± 1.18abc	29.08 ± 3.33h	–
	ICIPE 69	97.44 ± 0.72ab	91.42 ± 2.71a	3.84 (3.82–3.86)
	ICIPE 78	95.00 ± 3.00abcd	74.24 ± 4.07c	4.36 (4.33–4.39)
	ICIPE 81	97.82 ± 0.75a	65.84 ± 4.01de	4.52 (4.49–4.55)
*Beauveria bassiana*	ICIPE 279	87.25 ± 3.00d	13.5 ± 2.89i	–
	ICIPE 603	90.43 ± 3.30bcd	24.5 ± 3.61hi	–

Means within a column followed by the same letter are not significantly different following Student–Newman–Keuls (SNK) test at α = 0.05. ^1^ LT_50_ is the lethal time in days taken to kill 50% of the exposed adult flies; ^2^ FL is fiducial limit at 95%.

**Table 3:** Percentage eclosion (%) of *Zeugodacus cucurbitae* puparia at 5 days post-exposure to various conidial concentrations of the fungal isolates.

Concentration	Fungal Isolates		
	ICIPE 18	ICIPE 30	ICIPE 69
Control	91.00 ± 2.54		
1 × 10^6^	51.50 ± 13.19aA	39.00 ± 7.01aB	13.00 ± 2.39aC
1 × 10^7^	31.50 ± 12.09bA	25.00 ± 3.80bB	22.00 ± 5.03aB
1 × 10^8^	12.50 ± 1.76cA	10.00 ± 1.13cAB	8.00 ± 1.96bB

Means within a column followed by the same lowercase letter and within a row followed by the same uppercase letter are not significantly different following Student–Newman–Keuls (SNK) test at α = 0.05.

**Table 4:** Percentage mortality (%) of *Zeugodacus cucurbitae* adults eclosed from inoculated soil at different concentrations.

Conidial Concentration	Fungal Isolates		
	ICIPE 18	ICIPE 30	ICIPE 69
Control	06.68 ± 1.53		
1 × 10^6^	22.50 ± 3.33bA	34.38 ± 6.30bA	22.74 ± 14.95bA
1 × 10^7^	20.23 ± 8.17bA	51.22 ± 6.49aB	24.91 ± 8.76bA
1 × 10^8^	53.39 ± 6.07aAB	45.00 ± 2.89aB	74.29 ± 15.25aA

Means within a column followed by the same lowercase letter and within a row followed by the same uppercase letter are not significantly different following Student–Newman–Keuls (SNK) test at α = 0.05.

**Table 5:** Effect of cuelure on percentage conidial germination (%) of *Metarhizium anisopliae* ICIPE 69 over time.

Temperature	Days after Exposure									
	1		2		3		6		8	
	Cuelure	No Cuelure	Cuelure	No Cuelure	Cuelure	No Cuelure	Cuelure	No Cuelure	Cuelure	No Cuelure
18 °C	80.60 ± 0.79c	98.10 ± 0.80a	80.42 ± 1.41c	97.90 ± 0.37a	82.29 ± 1.42b	92.11 ± 0.62b	81.75 ± 2.95a	72.27 ± 2.95b	71.05 ± 2.85b	69.69 ± 1.49b
25 °C	92.09 ± 1.65b	98.46 ± 0.25a	89.69 ± 0.82b	96.26 ± 0.52a	83.44 ± 0.66b	95.81 ± 1.62a	77.19 ± 0.81a	93.88 ± 0.99a	68.91 ± 0.6b	88.93 ± 2.05a
30 °C	97.56 ± 0.96a	99.12 ± 0.08a	96.13 ± 0.92a	98.64 ± 0.17a	93.51 ± 0.46a	96.32 ± 0.45a	76.90 ± 2.88a	93.44 ± 0.27a	78.19 ± 1.51a	87.84 ± 0.38a

Means within a column followed by the same lowercase letter are not significantly different following Student–Newman–Keuls (SNK) test at α = 0.05.

**Table 6:** Effect of cuelure on mean conidial germ tube length (μm) of *Metarhizium anisopliae* ICIPE 69 over time.

Temperature	Days after Exposure									
	1		2		3		6		8	
	Cuelure	No Cuelure	Cuelure	No Cuelure	Cuelure	No Cuelure	Cuelure	No Cuelure	Cuelure	No Cuelure
18 °C	102.65 ± 5.13b	147.16 ± 12.82a	89.56 ±7.22b	119.78 ± 6.63b	56.53 ± 3.80b	110.61 ± 6.42a	37.92 ± 0.9c	83.05 ± 19.01a	27.15 ± 1.59b	75.52 ± 4.8b
25 °C	122.38 ± 2.88a	113.26 ± 2.28b	88.62 ± 6.05b	103.09 ± 0.92c	62.47 ± 4.64b	96.30 ± 3.02a	56.73 ± 1.43b	84.80 ± 2.04a	35.28 ± 3.52b	74.91 ± 4.79b
30 °C	123.19 ± 3.48a	145.33 ± 5.93a	119.59 ± 4.62a	140.64 ± 0.86a	103.13 ± 3.97a	97.25 ± 10.96a	91.55 ± 2.21a	96.78 ± 2.4a	85.59 ± 1.38a	91.48 ± 1.56a

Means within a column followed by the same lowercase letter are not significantly different following Student–Newman–Keuls (SNK) test at α = 0.05.

**Table 7:** Mortality of *Zeugodacus cucurbitae* after horizontally transmission of *Metarhizium anisopliae* ICIPE 69 inoculum through conspecifics flies.

	Mortality ± SE (%)	LT_50_ ^1^ (Days) (95% FL ^2^)
Male donor	100.00 ± 0.00a	2.93 (2.89–2.96)
Female donor	100.00 ± 0.00a	2.64 (2.61–2.68)
Male recipient	59.25 ± 5.92b	4.46 (4.38–4.53)
Female recipient	67.00 ± 4.49b	4.30 (4.24–4.53)

Means followed by the same letter are not significantly different following Student–Newman–Keuls (SNK) test at α = 0.05. ^1^ LT_50_ is the lethal time in days taken to kill 50% of the exposed adult flies; ^2^ FL is fiducial limit at 95%.

## Data Availability

The data presented in this study are available on request from the corresponding author.
